# Zebrafish excel in number discrimination under an operant conditioning paradigm

**DOI:** 10.1007/s10071-022-01602-y

**Published:** 2022-02-18

**Authors:** Angelo Bisazza, Maria Santacà

**Affiliations:** 1 Department of General Psychology, University of Padova, Padua, Italy; 2 Padua Neuroscience Center, University of Padova, Padua, Italy; 3 Department of Biology, University of Padova, Viale Giuseppe Colombo 3-Via Ugo Bassi 58/B, 35131 Padua, Italy

**Keywords:** Fish cognition, Numerical discrimination, Quantity discrimination, *Danio rerio*

## Abstract

Numerical discrimination is widespread in vertebrates, but this capacity varies enormously between the different species examined. The guppy (*Poecilia reticulata*), the only teleost examined following procedures that allow a comparison with the other vertebrates, outperforms amphibians, reptiles and many warm-blooded vertebrates, but it is unclear whether this is a feature shared with the other teleosts or represents a peculiarity of this species. We trained zebrafish (*Danio rerio*) to discriminate between numbers differing by one unit, varying task difficulty from 2 versus 3 to 5 versus 6 items. Non-numerical variables that covary with number, such as density or area, did not affect performance. Most fish reached learning criterion on all tasks up to 4 versus 5 discrimination with no sex difference in accuracy. Although no individual reached learning criterion in the 5 versus 6 task, performance was significant at the group level, suggesting that this may represent the discrimination threshold for zebrafish. Numerosity discrimination abilities of zebrafish compare to those of guppy, being higher than in some warm-blooded vertebrates, such as dogs, horses and domestic fowl, though lower than in parrots, corvids and primates. Learning rate was similar in a control group trained to discriminate between different-sized shapes, but zebrafish were slightly more accurate when discriminating areas than numbers and males were more accurate than females. At the end of the experiment, fish trained on numbers and controls trained on areas generalized to the reciprocal set of stimuli, indicating they had used a relational strategy to solve these tasks.

**Supplementary Information:**

The online version contains supplementary material available at 10.1007/s10071-022-01602-y.

## Introduction

Differences in cognitive abilities between species are generally believed to be associated with differences in the size and complexity of their nervous system (Deaner et al. 2007; Triki et al. 2021). However, there is growing evidence that natural selection can drive the evolution of sophisticated cognitive functions even in animals with small brains if these capacities confer a large fitness advantage. Clear examples of this phenomenon are the enhanced memory of food-storing birds, the homing capacities of pigeons, and the problem-solving capacities of some arthropods (Kamil and Gould 2008; Perry and Chittka 2019; Wallraff 2005).

Numerical abilities are widespread in all vertebrate groups studied so far but show great diversification, often even within the same taxonomic group. In some taxa of birds and mammals (notably corvids, parrots and primates), numerical competence rivals that of humans, whereas other mammalian and avian species show only rudimentary numerical abilities (e.g., Agrillo and Bisazza 2018; Nieder 2020; Pepperberg 2017). Although it has never been tested, it is commonly assumed that these differences are related to the different degrees of encephalization of the various taxa (e.g., Agrillo and Bisazza 2018; Davis and Memmott 1982; Skorupski et al. 2018). On the other hand, it is possible that for some species, numerical information is more important than it is for others. Numerical abilities have many different adaptive functions. For example, numerical discrimination allows dune snails (*Theba pisana)* to find a shelter to survive desiccation, female lions (*Panthera leo*) to estimate the strength of an opposing group, and banded killifish (*Fundulus diaphanous*) to select the social group that provides the best protection against predators (Bisazza and Gatto 2021; Hoare et al. 2004; McComb et al. 1994). We thus expect that different species have evolved different numerical capabilities in relation to the intensity of selection and the context in which they evolved. The limited number of species studied so far and the overrepresentation of some taxa (e.g., primates) precludes the possibility of testing the different hypotheses. Moreover, different taxonomic groups are often studied with different methods, thus preventing a direct comparison of the results obtained.

In teleosts, quantity discrimination has been investigated in several species (reviewed in Agrillo and Bisazza 2018; Agrillo et al. 2017) but, in most cases, they have been studied with the method of spontaneous preferences, allowing fish to choose between social groups of different sizes. Although these studies can yield valuable insight into teleosts’ social preferences, this approach usually cannot provide reliable information on their numerical abilities. In general, these experiments were unable to determine whether fish used numerical information or the other perceptual variables that covary with number; for example, the total activity of the shoals, the cumulative surface area occupied by fish, or the density of groups (Agrillo et al. 2007; Hager and Helfman 1991; Hoare et al. 2004). Indeed, studies that have attempted to experimentally control for non-numerical variables have frequently found teleosts’ discrimination of shoal numerosity dropped to chance level when the cumulative surface area and activity level of two stimulus shoals were equated (Agrillo et al. 2008b; Gómez-Laplaza and Gerlai 2013; Pritchard et al. 2001). This prevents a direct comparison with birds and mammals, which instead are generally studied using paradigms of forced two-choice appetitive operant conditioning with artificial stimuli for which non-numerical variables can be easily controlled. A few teleosts (i.e., redtail splitfin *Xenotoca eiseni*, zebrafish *Danio rerio*, Siamese fighting fish *Betta splendens*, mosquitofish *Gambusia affinis*, guppies *Poecilia reticulata*, and angelfish *Pterophyllum scalare*) have been investigated with fish-adapted appetitive conditioning paradigms. In these studies, fish proved able to discriminate stimuli controlled for non-numerical variables, although their performance was generally much lower than that of warm-blooded vertebrates (Agrillo et al. 2010). These procedures involved one or a few dozen reinforced trials and, again, these results are hard to compare with those of mammals and birds that normally undergo hundreds or even thousands of reinforced trials during each numerical experiment.

Only two species, the goldfish (*Carassius auratus*) and the guppy, have been studied so far with methods comparable to those of warm-blooded vertebrates, namely using a traditional paradigm of appetitive operant conditioning, an extensive training and stimuli controlled for non-numerical variables. Goldfish were extensively trained (1200 training trials) to discriminate different dot arrays (DeLong et al. 2017). Performance reached 90% accuracy in all ratios and was unaffected by non-numerical variable manipulation. Yet, the most difficult task used in this study was a 0.67 numerosity ratio discrimination (2 vs. 3 and 10 vs. 15), and whether goldfish could achieve the higher ratio discriminations (e.g., 0.80 or 0.90) used in studies on higher vertebrates remains unsettled (Beran 2008; Emmerton and Delius 1993; Tomonaga 2008).

In two separate studies, it was found that guppies discriminate numbers that differ by 1 unit up to 4 versus 5 items, corresponding to a 0.80 numerosity ratio (Bisazza et al. 2014; Gatto et al. 2021). As humans and some other mammals and birds (Mix et al. 2002; Rugani et al. 2013; Tomonaga 2008), guppies can integrate numerical and non-numerical information to obtain a more accurate estimate but, if prevented from accessing the latter, they can discriminate based on the sole numerical information (Gatto et al. 2021). The numerical acuity shown by guppies is lower compared to humans, non-human primates and crows but exceeds that observed in cartilaginous fishes, amphibians, reptiles and various warm-blooded vertebrates such as dogs (*Canis lupus familiaris*), horses (*Equus caballus*) and domestic chicken (*Gallus gallus*) (reviewed in Agrillo and Bisazza 2018). Excellent capacities of guppy compared to the other vertebrate species have been confirmed for other numerical tasks (Lucon-Xiccato et al. 2017; Miletto Petrazzini et al. 2015a, b; Miletto Petrazzini et al. 2015a, b).

It has been suggested that guppy’s excellent numerical abilities may be because they are crucial in many contexts. Quantificational abilities can potentially decrease predation risk, aid foraging decisions, reduce the costs of sexual conflict in females and increase mating success in males (Agrillo et al. 2006; Day et al. 2001; Lindström and Ranta 1993; Lucon-Xiccato et al. 2016). In all these cases, guppies are required to identify and count the exact number of objects of a given category (males, females, competitors or preys) present in their neighbourhood, a task that cannot be accomplished by simply estimating the density or the total surface occupied by the items. It is also possible that teleosts in general are endowed with excellent numerical skills. In recent years, research has found that teleosts possess sophisticated cognitive abilities, despite their brains being much smaller than those of warm-blooded vertebrates are (Bshary and Brown 2014). Molecular data have shown that after the separation from the line of fish that gave rise to land vertebrates, teleosts enjoyed a great evolutionary advantage due to a whole genome duplication. This event made available thousands of new genes, the raw material on which selection could act; hence, favouring the rapid evolution of novel morpho-physiological and behavioural traits and the colonization of all aquatic habitats (Ravi and Venkatesh 2018; Schartl et al. 2013).

At present, the hypothesis that the numerical abilities of teleosts are comparable to those of higher vertebrates and the hypothesis that the numerical abilities of guppies reflect unique selective pressures are equally likely. It is therefore critical to study other teleost species using procedures that allow direct interspecific comparison. Zebrafish is an emerging model for studying brain functions, and many of these studies require detailed knowledge of its cognitive abilities. Guppy and zebrafish are phylogenetically distant, belonging, respectively, to Acanthopterygians and Ostariophysians, the two large clades that constitute the large majority of teleost species and that have evolved separately for approximately 220 million years (Steinke et al. 2006).

Information on zebrafish’s numerical capacities is scarce. The only study in which zebrafish was examined using stimuli controlled for non-numerical variables used a place learning paradigm in which a food reward was repeatedly released close to one of two numerical quantities, and, after this training phase, the time subjects spent near each stimulus was measured in probe trials (Agrillo et al. 2012). In this study, zebrafish performed significantly worse than the other species investigated did. On the other hand, in the same study, zebrafish also showed a poor performance in a control task consisting of shape discrimination and it was suggested that this species might be less efficient at learning with this kind of conditioning paradigm. Two studies recently examined zebrafish’s ability to select the larger of two social groups (Potrich et al. 2015; Seguin and Gerlai 2017). Both studies indicate reduced ability of zebrafish to discriminate compared to other species examined, but methodological differences between studies or interspecific differences in social behaviour could also explain these results (Gimeno et al. 2016; Lucon-Xiccato et al. 2017).

In this study, we examined the ability of male and female zebrafish to discriminate the larger or the smaller of two numerosities using an appetitive conditioning paradigm and stimuli controlled for the main non-numerical variables. Different methods often yield different results (Bánszegi et al. 2016; Gatto et al. 2021; Howard et al. 2019), and interspecific differences in numerical abilities might be attributable to differences in the methodologies used in the different studies. Here, we adopted the same procedure employed recently in a study in guppy so that a direct comparison with this species was possible (Gatto et al. 2021). Subjects were given a series of discriminations of increasing difficulty (from 2 versus 3 items to 5 versus 6 items) to determine their numerical acuity threshold. If capacities shown by guppies are typical of teleosts, then we expect good performances of zebrafish in numerical discrimination. Some studies seem to suggest that zebrafish differs from other species in discriminative learning abilities (Agrillo et al. 2012; Gatto et al. 2020). For this reason, a control group was evaluated in another quantitative task, the discrimination of two identical shapes differing in area. Using a spontaneous preference paradigm, zebrafish have previously shown to accurately discriminate between sizes (Santacà et al. 2020a, b). In the control experiment, we used the same procedure and the same ratios proposed in the numerical experiment.

Numerical discrimination tasks can be solved in two ways: by learning the absolute values of the stimuli or developing the relational concept of larger and smaller. To gain insight into the strategy used by zebrafish, at the end of the experiments, fish trained on numbers were tested in unreinforced trials that featured figures with different areas, whereas those trained to discriminate areas were given the choice of two numerosities. If zebrafish had developed a relational strategy, then we would expect them to generalize the learned rule to the new class of stimuli.

## Materials and methods

### Subjects and animal housing

Sixteen adult zebrafish (8 males and 8 females) were used in the numerical discrimination experiment. Sixteen additional fish (8 males and 8 females) were used as controls and trained in another quantitative task, the discrimination of two identical shapes differing in area. All fish were experimentally naïve. Zebrafish were bought from a local supplier and were maintained for at least 1 month in a large-outbred stock (approximately 200 adults) in a 400-L plastic tank with a gravel bottom, vegetation and two biomechanical filters. The water temperature was maintained at 26 ± 1 °C with a 12:12 h light/dark photoperiod. Zebrafish were fed commercial food flakes (Aqua Tropical, Padovan) in the morning and live brine shrimp (*Artemia salina*) in the afternoon. After participating in the experiment, subjects were moved to a specific stock tank and kept for breeding purposes.

### Apparatus

**Fig. 1:**
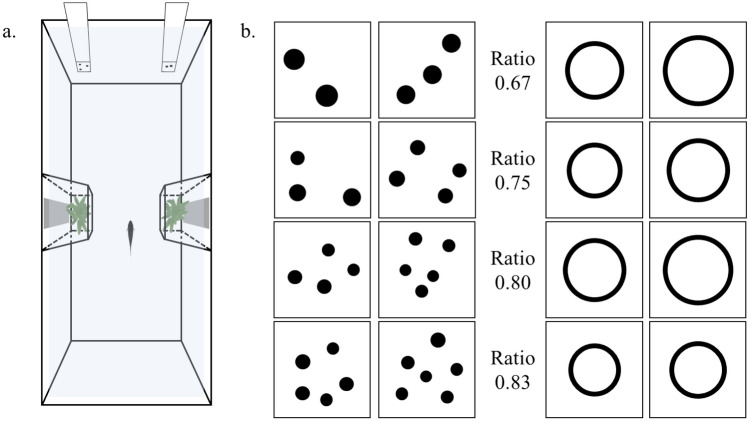
Experimental apparatus and stimuli. **a** Three-dimensional aerial view of the apparatus. The tank was subdivided in a central hourglass-shaped testing compartment and two lateral compartments that housed natural plants and a mirror. Examples of stimuli used in **b** numerical discrimination and **c** discrimination of areas

Each subject was individually maintained for the duration of the experiment in a glass tank (22 × 50 × 32 cm) filled with 28 L of water (see Gatto et al. 2021; Santacà et al. 2021 for more details). Two trapezoidal structures (10 × 6 × 32 cm) made with transparent acetate sheets subdivided the tank in an hourglass-shaped testing compartment and two small lateral compartments (Fig. 1a). To increase the visibility of the stimuli, the inside of both short walls was covered with white polyester sheets. The two long sides were covered with opaque green plastic sheets. The tank was provided with natural loose gravel and plants that were confined in two lateral compartments. Additionally, to minimize stress due to social isolation (Miletto Petrazzini et al. 2012), each lateral compartment was provided with a mirror and a recirculating water system put each tank in olfactory communication with a large aquarium containing a group of conspecifics (see Supplementary Methods). Subjects were tested in eighteen identical apparatuses placed in a dark room. Tanks were lit by fluorescent lamps with a 12:12 h light/dark photoperiod. Water temperature in the experimental tanks was maintained at 26 ± 1 °C. All trials were recorded with video cameras placed above each tank.

### Stimuli

Stimuli were made with Adobe Illustrator CC 2019 and printed on laminated white cards (3 × 3 cm). Each card was affixed to a transparent Plexiglas panel (3.5 × 15 cm) that was hung on the wall of the tank.

*Numerical discrimination*. Stimuli consisted of sets of black dots (diameter range 0.75–0.95 cm) on a white background (Fig. 1b; see also Supplementary Material). We presented four different numerical ratios: 2 versus 3 (ratio 0.67), 3 versus 4 (ratio 0.75), 4 versus 5 (ratio 0.80), and 5 versus 6 (ratio 0.83). As other vertebrates, fish can discriminate two quantities of items using non-numerical cues that co-vary with number, such as the cumulative surface area of the items, the space occupied by the array or “convex hull” (measured drawing the smallest polygon enclosing all dots in an array) and the density of items (Gómez-Laplaza and Gerlai 2013; Lucon-Xiccato et al. 2015). We used 24 different pairs of stimuli for each ratio that were controlled for the three above-mentioned non-numerical variables. As a side effect of equating cumulative surface area, the larger set of items tends to contain smaller than average dots. If stimuli are controlled to 100% for area, fish could use the size of dots in place of numerical information to solve the task. For this reason, the ratio between the cumulative surface areas of the smaller over the larger set of stimuli was 76–85% in one-third, 86–95% in one-third, and 96–105% in the final one-third. The control for cumulative surface area also produced a partial control for cumulative contour length. The ratio between the cumulative contour lengths of the smaller over the larger set was similar to that of areas, ranging between 79 and 105%. Convex hull and density are inversely related and cannot be controlled for simultaneously. Therefore, we equated convex hull in half of the trials and the density in the other half. Prior to beginning the numerical task, subjects were pre-trained using a very easy discrimination, 3 versus 12 items (18 different pairs), with same characteristics described above but not controlled for the cumulative surface area (see Supplementary Material).

*Continuous quantity discrimination* The stimuli of the control group consisted of two identical shapes, namely outlined black circles, differing in area by the same ratios proposed in the numerical experiment (0.25, 0.67, 0.75, 0.80, and 0.83; Fig. 1b). We used a circular shape to allow easier comparison with an earlier study in which size discrimination was studied with a spontaneous preference paradigm (Santacà et al. 2020a, b), i.e. exploiting the natural tendency of zebrafish to pass through the largest hole. Five subjects were also tested in the 0.86 ratio discrimination because they reached the learning criterion in the 0.83 task. For each ratio, we used six different pairs of stimuli in which we systematically varied the area of the two circles, whereas the ratio was kept constant. The diameter of the circles varied from 1 to 2.50 cm. The ranges of the six versions partially overlapped and, therefore, the same circle could be the larger in one pair and the smaller in another one.

### Procedure

The training procedure was the same for the numerical discriminations and for controls trained on continuous quantity except for the type of stimuli used. Following the protocol of previous studies (Gatto et al. 2021; Santacà et al. 2021), each subject underwent a habituation phase and a training phase. During the 2-day habituation phase, subjects became familiar with the experimental apparatus. On both days, zebrafish were fed four times releasing live brine shrimp nauplii (*Artemia salina*) with a 3 ml plastic Pasteur pipette, alternating positions near the two short sides of the apparatus.

Before the actual discrimination experiment, both experimental and control subjects underwent a pre-training consisting of an easy discrimination task (respectively, 3 versus 12 items not controlled for the cumulative surface area and two circles differing by a 0.25 ratio). Pre-training consisted of 12 trials per day for a maximum of 12 consecutive days. Subjects were admitted to the experiment if they reached one of the two learning criteria (see below).

During the experiment, subjects underwent a series of discriminations of increasing difficulty (from 2 versus 3 items to 5 versus 6 items). In each task, they performed six trials in the morning and six in the afternoon, with a 90-min interval between the sessions and a 15-min interval between two consecutive trials. The training phase lasted a maximum of 10 consecutive days for each ratio. Half of the subjects (four females and four males) were trained to select the larger numerosity; half (four females and four males) were trained to select the smaller numerosity. In each trial, a subject was presented with two numerosities by simultaneously inserting the two transparent panels with stimuli on the same short wall of the tank, while the subject was near the opposite side of the tank. The left/right position of the larger quantity and the short side of the tank on which the stimuli were presented were counterbalanced using a semi-random sequence.

A choice was recorded when the subject approached (swam at less than 1 body length) one stimulus. To assess reliability of this measure, one-third of the video-recorded trials of each ratio for each subject was analysed by a second observer who was blind to the experimental hypotheses. The reliability for both experiments was found to be very high (see Supplementary Material). When the subjects chose the correct stimulus, they were given a food reward (i.e., a drop of live brine shrimp nauplii) and, simultaneously, the wrong stimulus was removed. If they approached the wrong stimulus first, no food reward was given and both stimuli were removed simultaneously.

We considered two learning criteria. The primary learning criterion was defined as at least 75% correct choices (18/24 trials) over 2 consecutive days (statistically significant at the binomial test). The secondary learning criterion was defined as a frequency of at least 60% correct choices over the whole training (72/120 trials, statistically significant at the binomial test). If they failed to reach one of the two criteria within 120 trials, the experiment ended. If subjects met one of the two learning criteria, they were admitted to the following, more difficult discrimination.

*Absolute versus relational discrimination* After completing the training phase, experimental fish were investigated to assess whether they had solved the numerical task using a relational strategy (i.e., developing the relational concept of larger and smaller) or by learning to respond to a specific numerosity. Subjects underwent a further test phase in which they were presented with the 3 versus 4 numerical contrast (with the same procedure as previous training trials) intermingled with probe trial (without reward) in which we presented two circles differing in area by a 0.75 ratio. Zebrafish of the control group underwent a similar test, and they were tested in standard trials with an area discrimination (0.75 ratio) intermingled with probe trials in which we presented a 3 versus 4 numerical contrast. The procedure for unreinforced trials was identical to that of a standard trial except that fish were observed for 2 min after the insertion of the stimuli and no food reinforcement was delivered. Fish received 12 trials (eight standard trials and four extinction trials) per day, for 2 consecutive days. We used a pseudorandom procedure and unreinforced trials were never at the beginning or the end of the sequence and were never consecutive. The left/right position of the larger quantity in both standard trials and probe trials was counterbalanced using a semi-random sequence. We recorded the first choice in both standard and probe trials. In addition, in probe trials we made a measurement on videorecordings of the percentage of time spent near each stimulus (within one body length) in the two minutes of test (see Supplementary Material for more details).

### Statistical analysis

During the experiment, we used the two learning criteria specified above to conclude a task and admit a subject to the subsequent task. At the end of the experiments, we analysed the effects of sex, inter-individual differences, ratio, and control of continuous variables using generalized linear models fitted with subjects’ accuracy (number of correct and incorrect trials) as independent variable. Task achievement was determined at the group level by analysing the individual proportion of correct responses with one-sample *t* tests. Normality of data was checked using the Shapiro–Wilk test. Task achievement at the individual level was determined using binomial tests on all trials of the task.

Analyses were performed in R version 4.0.5 (The R Foundation for Statistical Computing). Data of the pre-training trials (in which the cumulative surface was not controlled) were not included in the analysis. We first conducted two separate analyses for the numerical discrimination experiment and for the continuous quantity discrimination control experiment. For both we performed a generalized mixed-effects model for binomial distributions (GLMM) with the reinforced stimulus, the training session, the sex and the ratio as fixed effects, and the individual ID as a random effect. We checked for the presence of inter-individual differences in numerical discrimination by comparing a model with individual ID as a random effect to a model without such factor (ANOVA function of the Car package; Fox and Weisberg 2019); the 5 vs 6 discrimination was omitted from this analysis since only some subjects were tested in this task. We assessed inter-individual differences in continuous quantity discrimination in the same way.

*Factors influencing numerical discrimination* We performed a separate GLMM to assess whether the performance differed between the three levels of control for cumulative surface area or between the stimuli controlled for density and those controlled for convex hull. Due to collinearity between cumulative surface area and cumulative contour length, we performed a separate GLMM to assess whether the performance differed between three levels of control for cumulative contour length. As these analyses yielded a non-significant effect of non-numerical variables, we performed Bayesian reanalysis of the null results to evaluate the strength of evidence in favour of the null hypothesis (Dienes 2014; Harms and Lakens 2018; Hoekstra et al 2018). In three separate analyses (for cumulative surface area, for cumulative contour length and for convex hull vs. density control, respectively), we compared the relative strength of the model including the factor and the model without the factor. We calculated the approximate Bayes factor with the generalTestBF function of the BayesFactor R package. For the cumulative surface area and the cumulative contour length, after checking for normality (Shapiro–Wilk test, *p* > 0.05), we performed a *t* test analysis on the sole block of stimuli controlled on average to 100% (block 96–105% control) and on a subsample of trials in which stimuli were controlled 100–105% (16 out of 96 pairs of stimuli).

*Comparison with guppy* To compare the performance of zebrafish in the numerical discrimination tasks with the data obtained with the same procedure in the guppy (Gatto et al. 2021), we performed a GLMM fitting the species and the ratio as fixed effects and the individual ID nested within the species as a random effect. Because Gatto et al. (2021) trained only female guppies, a second GLMM was performed considering only the data of female zebrafish.

*Comparison between numerical and continuous quantity discriminations* We performed an overall GLMM analysis to compare the performance in the two tasks. We fitted the model with the type of discrimination, the training session and the ratio as fixed effects, and the individual ID nested within the discrimination as a random effect.

*Absolute versus relational discrimination* We performed two separate analyses for the frequency of first choices and for the time spent near the previously reinforced quantity in probe trials. All data were normally distributed (Shapiro–Wilk test, *p* > 0.05); thus, we performed one-sample *t* tests (chance level = 0.5) for both measures. We performed a GLMM to assess whether fish trained on numbers and controls trained on areas differed in accuracy of probe trials. For numerical probe trials, we also performed a GLMM to assess the effect of control for cumulative surface area.

## Results

### Numerical discrimination

**Fig. 2:**
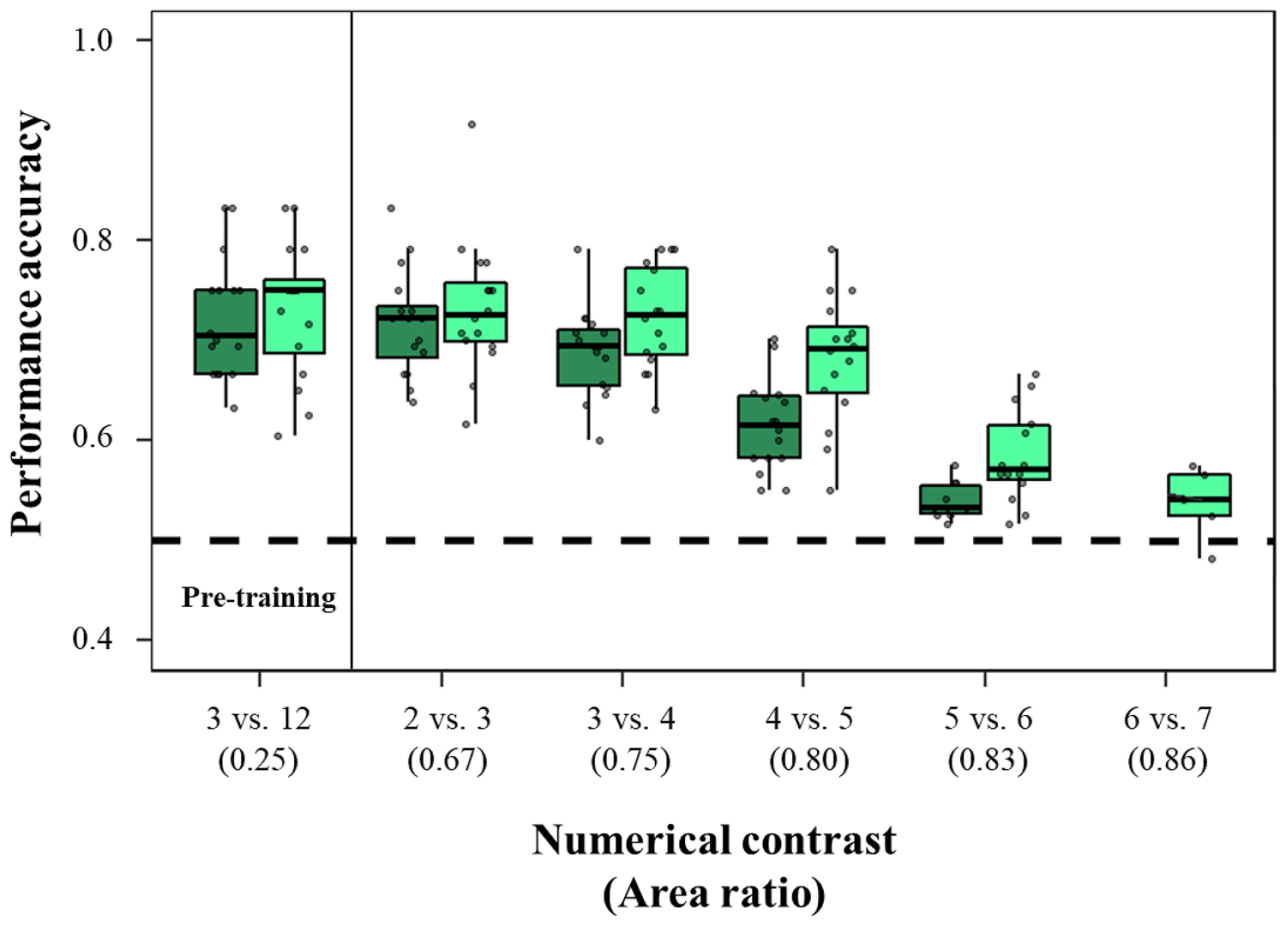
Results. Comparison of the zebrafish performance in numerical discrimination (dark green) and area discrimination (light green) in all contrasts tested. In the pre-training, subjects underwent a 3 versus 12 discrimination (not controlled for cumulative surface area) and a 0.25 area ratio discrimination, respectively. The boxplots report median, first quartile, third quartile, ranges, and outliers (data points 1.5 interquartile ranges smaller than the first quartile or greater than the third quartile). The dotted line represents chance performance (50% correct responses). Dots represent individual mean proportion of choices for the reinforced quantity

All 16 subjects reached the primary learning criterion (18/24 correct choices in two consecutive days) during the pre-training phase (Table 1; Fig. 2). All 16 subjects also reached the primary learning criterion in the 2 versus 3 and in 3 versus 4 discrimination. Nine out of 16 subjects (4 females and 5 males) reached the primary learning criterion in the 4 versus 5 discrimination. One additional male reached the secondary learning criterion. None of these 10 subjects achieved the 5 versus 6 discrimination according to the learning criteria. However, performance in the 5 versus 6 discrimination was significant at the group level (see Table 1).

**Table 1:** Individual and group performance of zebrafish in the numerical discrimination

Subjects	Sex	Reinforced quantity	Pre-training (3 versus 12)	2 versus 3	3 versus 4	4 versus 5	5 versus 6
1	♀	Larger	75.00 ± 8.33% 27/36 *p* = 0.004	69.44 ± 17.35% 25/36 *p* = 0.029	79.17 ± 17.68% 19/24 *p* = 0.007	55.00 ± 10.54% 66/120 *p* = 0.315	N/A
2	♀	Larger	66.67 ± 10.21% 40/60 *p* = 0.013	66.67 ± 10.54% 48/72 *p* = 0.006	60.00 ± 16.03% 36/60 *p* = 0.155	58.33 ± 6.80% 70/120 *p* = 0.082	N/A
3	♀	Larger	75.00 ± 23.57% 18/24 *p* = 0.023	75.00 ± 6.80% 36/48 *p* < 0.001	70.00 ± 4.56% 42/60 *p* = 0.003	58.33 ± 11.11% 70/120 *p* = 0.082	N/A
4	♀	Larger	66.67 ± 22.05% 24/36 *p* = 0.065	72.92 ± 10.49% 35/48 *p* = 0.002	63.54 ± 14.73% 61/96 *p* = 0.010	63.89 ± 10.09% 46/72 *p* = 0.024	51.67 ± 13.49% 62/120 *p* = 0.784
5	♀	Smaller	83.33 ± 0.00% 20/24 *p* = 0.002	75.00 ± 0.00% 18/24 *p* = 0.023	68.33 ± 6.97% 41/60 *p* = 0.006	64.29 ± 9.27% 54/84 *p* = 0.012	54.17 ± 11.95% 65/120 *p* = 0.411
6	♀	Smaller	70.83 ± 10.76% 34/48 *p* = 0.006	95.83 ± 5.89% 23/24 *p* < 0.001	68.75 ± 12.50% 33/48 *p* = 0.013	61.11 ± 14.59% 44/72 *p* = 0.076	53.33 ± 9.78% 64/120 *p* = 0.523
7	♀	Smaller	83.33 ± 11.79% 20/24 *p* = 0.002	72.22 ± 9.62% 26/36 *p* = 0.011	69.44 ± 20.97% 25/36 *p* = 0.029	58.33 ± 5.83% 70/120 *p* = 0.082	N/A
8	♀	Smaller	70.00 ± 15.14% 42/60 *p* = 0.003	72.92 ± 7.98% 35/48 *p* = 0.002	72.22 ± 4.81% 26/36 *p* = 0.011	61.90 ± 15.85% 52/84 *p* = 0.038	53.33 ± 9.78% 64/120 *p* = 0.523
9	♂	Larger	75.00 ± 8.33% 27/36 *p* = 0.004	66.67 ± 8.33% 40/60 *p* = 0.013	70.83 ± 13.69% 51/72 *p* < 0.001	56.67 ± 9.46% 68/120 *p* = 0.171	N/A
10	♂	Larger	63.33 ± 11.18% 38/60 *p* = 0.052	79.17 ± 5.89% 19/24 *p* = 0.007	69.44 ± 9.62% 25/36 *p* = 0.029	69.44 ± 10.09% 50/72 *p* = 0.001	55.83 ± 9.66% 67/120 *p* = 0.235
11	♂	Larger	75.00 ± 11.79% 18/24 *p* = 0.023	68.75 ± 7.98% 33/48 *p* = 0.013	64.58 ± 20.83% 31/48 *p* = 0.059	62.04 ± 10.30% 67/108 *p* = 0.016	55.83 ± 11.15% 67/120 *p* = 0.235
12	♂	Larger	69.44 ± 12.73% 25/36 *p* = 0.029	65.00 ± 12.36% 39/60 *p* = 0.027	70.83 ± 12.64% 51/72 *p* < 0.001	64.58 ± 10.68% 62/96 *p* = 0.006	52.50 ± 14.19% 63/120 *p* = 0.648
13	♂	Smaller	69.44 ± 12.73% 25/36 *p* = 0.029	77.78 ± 12.73% 28/36 *p* = 0.001	65.28 ± 12.27% 47/72 *p* = 0.195	60.00 ± 13.49% 72/120 *p* = 0.035	57.50 ± 8.29% 69/120 *p* = 0.120
14	♂	Smaller	66.67 ± 13.61% 32/48 *p* = 0.029	63.89 ± 20.97% 23/36 *p* = 0.133	72.22 ± 12.73% 26/36 *p* = 0.011	64.81 ± 12.34% 70/108 *p* = 0.003	52.50 ± 8.83% 63/120 *p* = 0.648
15	♂	Smaller	79.17 ± 17.68% 19/24 *p* = 0.007	72.22 ± 17.35% 26/36 *p* = 0.011	65.63 ± 11.30% 63/96 *p* = 0.003	70.24 ± 8.13% 59/84 *p* < 0.001	53.33 ± 10.54% 64/120 *p* = 0.523
16	♂	Smaller	66.67 ± 18.63% 40/60 *p* = 0.013	83.33 ± 11.79% 20/24 *p* = 0.002	71.67 ± 15.14% 43/60 *p* = 0.001	55.00 ± 7.03% 66/120 *p* = 0.315	N/A
**Overall**			72.22 ± 6.05% *t*_15_ = 47.731 *p* < 0.001	71.76 ± 5.25% *t*_15_ = 54.589 *p* < 0.001	68.86 ± 4.44% *t*_15_ = 62.043 *p* < 0.001	61.49 ± 4.57% *t*_15_ = 53.824 *p* < 0.001	53.99 ± 1.82% *t*_9_ = 93.470 *p* < 0.001

In each cell, we reported the percentage of correct responses (mean ± standard deviation) and the number of correct responses / number of total choices for individual fish. The *p* value was calculated with the binomial test for individual analysis and with one-sample *t* tests for group analyses

The overall analysis revealed a significant improvement in subjects’ accuracy over training session (GLMM: $\chi_{1}^{2}$  = 18.976, *p* < 0.001), and a significant decrease in subjects’ accuracy when increasing the ratio between numerosities ($\chi_{1}^{2}$ = 65.312, *p* < 0.001). Males and females did not significantly differ ($\chi_{1}^{2}$ = 0.032, *p* = 0.868), and we found no difference between zebrafish trained to select the larger numerosity or the smaller numerosity ($\chi_{1}^{2}$ = 1.792, *p* = 0.181). No interaction was statistically significant (all *p* values > 0.080). Visual inspection of the data (Fig. 2) suggests that decrease in subjects’ accuracy with increasing ratio was not linear. In particular, accuracy appeared to be constant up to 3 versus 4 discrimination whereas it decreased with increasing ratio for the subsequent tasks. A GLMM confirmed no variation in accuracy ($\chi_{1}^{2}$ = 0.316, *p* = 0.574) between the first three tasks (3 versus 12, 2 versus 3, 3 versus 4) and a significant negative linear trend from the 3 versus 4 task onward (3 versus 4, 4 versus 5, 5 versus 6; $\chi_{1}^{2}$ = 15.451, *p* < 0.001).

Zebrafish performance did not differ between the three levels of control for cumulative surface area ($\chi_{2}^{2}$ = 2.884, *p* = 0.236) nor between the stimuli controlled for density and those controlled for convex hull ($\chi_{1}^{2}$ = 0.033, *p* = 0.856). The approximate Bayes factors indicated that GLMM models without the effect of the cumulative surface area or without the effect of the density and convex hull were, respectively, 47 and 76 more likely to explain the performance of the subjects than the models with such effects. Moreover, we found a significant preference for the reinforced stimulus even considering only the block with greater percentage (96–105%) of correction for cumulative surface areas (one sample *t* test, *t*_15_ = 9.327, *p* < 0.001) or the subsample of trials with correction between 100 and 105% (one sample *t* test, *t*_15_ = 12.175, *p* < 0.002).

Control for cumulative surface also produces a partial control for cumulative contour length. A GLMM analysis showed that performance did not differ between three levels of control for cumulative contour length (levels: 79–85%, 86–95% and 96–105%; $\chi_{2}^{2}$ = 1.598, *p* = 0.450). The approximate Bayes factor indicated that GLMM model without the effect of the cumulative contour length was 51 more likely to explain the performance of the subjects than the model with such effect. Moreover, we found a significant preference for the reinforced stimulus even considering only those trials in which the contour ratio was between 96 and 105% (one sample *t* test, *t*_15_ = 7.152, *p* < 0.001) or the subsample of trials with correction between 100 and 105% (one sample *t* test, *t*_15_ = 8.975, *p* < 0.001).

The comparison of the log-likelihoods of models with and without the random effect revealed no inter-individual difference in numerical discrimination (*p* = 1.000). The same result was obtained analysing each ratio separately (all *p *values > 0.128).

### Comparison with guppies

When comparing numerical discrimination in zebrafish and guppies (data from Gatto et al. 2021) we found that the two species did not differ in performance (GLMM: $\chi_{1}^{2}$ = 0.977, *p* = 0.323). Subjects’ accuracy significantly decreased when increasing the ratio between numerosities ($\chi_{1}^{2}$ = 41.976, *p* < 0.001). The interaction species × ratio was not statistically significant ($\chi_{1}^{2}$ = 2.387, *p* = 0.122). The same results were obtained considering only the females trained on the larger numerosity as in the experiment on guppy.

### Continuous quantity discrimination

In the pre-training, out of 16 subjects reached the primary learning criterion of 18/24 correct choices in two consecutive days (Table 2; Fig. 2). All 16 subjects reached the primary learning criterion in the 0.67 and in the 0.75 discriminations. Thirteen out of 16 subjects (5 females and 8 males) reached the primary learning criterion in the 0.80 discrimination. One additional female reached the secondary learning criterion. Two out of 14 subjects (both males) reached the primary learning criterion in the 0.83 discrimination. Three additional subjects (one female and two males) reached the secondary learning criterion. None of these five subjects achieved the 0.86 discrimination according to the learning criteria. Overall, subjects’ accuracy in the 0.86 area discrimination was greater than that expected at chance level (see Table 2).

**Table 2:** Individual and group performance of zebrafish in the continuous quantity (area) discrimination

Subjects	Sex	Reinforced quantity	Pre-training (0.25)	0.67	0.75	0.80	0.83	0.86
1	♀	Larger	75.00 ± 11.79% 18/24 *p* = 0.023	69.44 ± 9.62% 25/36 *p* = 0.029	79.17 ± 17.68% 19/24 *p* = 0.007	72.92 ± 4.17% 35/48 *p* = 0.002	56.67 ± 9.46% 68/120 *p* = 0.171	N/A
2	♀	Larger	79.17 ± 5.89% 19/24 *p* = 0.007	79.17 ± 5.89% 19/24 *p* = 0.007	79.17 ± 5.89% 19/24 *p* = 0.007	79.17 ± 5.89% 19/24 *p* = 0.007	60.83 ± 7.91% 73/120 *p* = 0.022	57.50 ± 13.86% 69/120 *p* = 0.120
3	♀	Larger	83.33 ± 11.79% 20/24 *p* = 0.002	70.00 ± 12.64% 42/60 *p* = 0.003	68.06 ± 12.27% 49/72 *p* = 0.003	55.00 ± 7.03% 66/120 *p* = 0.315	N/A	N/A
4	♀	Larger	69.44 ± 12.73% 25/36 *p* = 0.029	91.67 ± 11.79% 22/24 *p* < 0.001	79.17 ± 17.68% 19/24 *p* = 0.007	63.89 ± 8.33% 69/108 *p* = 0.005	54.17 ± 10.58% 65/120 *p* = 0.411	N/A
5	♀	Smaller	60.42 ± 11.31% 87/144 *p* = 0.015	65.48 ± 11.21% 55/84 *p* = 0.006	63.10 ± 10.60% 53/84 *p* = 0.021	60.83 ± 11.82% 73/120 *p* = 0.022	51.67 ± 10.24% 62/120 *p* = 0.784	N/A
6	♀	Smaller	75.00 ± 11.79% 18/24 *p* = 0.023	75.00 ± 0.00% 18/24 *p* = 0.023	69.44 ± 16.39% 50/72 *p* = 0.001	65.00 ± 10.87% 39/60 *p* = 0.027	55.83 ± 7.91% 67/120 *p* = 0.235	N/A
7	♀	Smaller	75.00 ± 11.79% 18/24 *p* = 0.023	72.22 ± 12.73% 26/36 *p* = 0.011	66.67 ± 18.00% 32/48 *p* = 0.029	70.83 ± 17.35% 34/48 *p* = 0.006	52.50 ± 7.91% 63/120 *p* = 0.648	N/A
8	♀	Smaller	62.50 ± 15.96% 30/48 *p* = 0.111	61.67 ± 17.28% 37/60 *p* = 0.092	72.22 ± 17.35% 26/36 *p* = 0.011	59.17 ± 8.29% 71/120 *p* = 0.055	N/A	N/A
9	♂	Larger	83.33 ± 23.57% 20/24 *p* = 0.002	77.78 ± 12.73% 28/36 *p* = 0.001	75.00 ± 8.33% 27/36 *p* = 0.004	75.00 ± 8.33% 27/36 *p* = 0.004	66.67 ± 12.60% 64/96 *p* = 0.001	52.50 ± 11.82% 63/120 *p* = 0.648
10	♂	Larger	66.67 ± 15.21% 32/48 *p* = 0.029	68.75 ± 7.98% 33/48 *p* = 0.013	72.92 ± 14.23% 35/48 *p* = 0.002	69.05 ± 9.27% 58/84 *p* < 0.001	56.67 ± 7.66% 68/120 *p* = 0.171	N/A
11	♂	Larger	72.92 ± 14.23% 35/48 *p* = 0.002	77.78 ± 12.73% 28/36 *p* = 0.001	77.08 ± 10.49% 37/48 *p* < 0.001	75.00 ± 11.79% 36/48 *p* < 0.001	56.67 ± 9.46% 68/120 *p* = 0.171	N/A
12	♂	Larger	79.17 ± 5.89% 19/24 *p* = 0.007	75.00 ± 14.43% 27/36 *p* = 0.004	72.92 ± 14.23% 35/48 *p* = 0.003	69.44 ± 17.21% 50/72 *p* = 0.001	65.48 ± 12.20% 55/84 *p* = 0.006	56.67 ± 7.66% 68/120 *p* = 0.171
13	♂	Smaller	65.00 ± 16.03% 39/60 *p* = 0.027	70.83 ± 10.76% 34/48 *p* = 0.006	66.67 ± 10.21% 40/60 *p* = 0.013	70.24 ± 10.60% 59/84 *p* < 0.001	64.17 ± 6.86% 77/120 *p* = 0.002	54.17 ± 8.10% 65/120 *p* = 0.411
14	♂	Smaller	75.00 ± 8.33% 27/36 *p* = 0.004	72.92 ± 14.23% 35/48 *p* = 0.002	68.75 ± 10.49% 33/48 *p* = 0.013	66.67 ± 9.62% 32/48 *p* = 0.029	57.50 ± 7.30% 69/120 *p* = 0.120	N/A
15	♂	Smaller	75.00 ± 8.33% 27/36 *p* = 0.004	70.83 ± 10.76% 34/48 *p* = 0.006	70.83 ± 10.76% 34/48 *p* = 0.006	67.86 ± 7.50% 57/84 *p* = 0.001	57.50 ± 8.29% 69/120 *p* = 0.120	N/A
16	♂	Smaller	71.67 ± 18.26% 43/60 *p* = 0.001	75.00 ± 0.00% 18/24 *p* = 0.023	77.78 ± 17.35% 28/36 *p* = 0.001	70.24 ± 8.13% 59/84 *p* < 0.001	61.67 ± 5.83% 74/120 *p* = 0.013	48.33 ± 10.24% 58/120 *p* = 0.784
**Overall**			73.04 ± 6.81% *t*_15_ = 42.878 *p* < 0.001	73.35 ± 6.72% *t*_15_ = 43.642 *p* < 0.001	72.44 ± 5.11% *t*_15_ = 56.711 *p* < 0.001	68.14 ± 6.29% *t*_15_ = 43.329 *p* < 0.001	58.44 ± 4.67% *t*_13_ = 46.794 *p* < 0.001	53.75 ± 4.24% *t*_4_ = 25.332 *p* < 0.001

In each cell, we reported the percentage of correct responses (mean ± standard deviation) and the number of correct responses/number of total choices for individual fish. The *p* value was calculated with the binomial test for individual analysis and with one-sample *t* tests for group analyses

The overall analysis revealed a significant improvement in subjects’ accuracy over training session (GLMM: $\chi_{1}^{2}$ = 16.543, *p* < 0.001), and a significant decrease in subjects’ accuracy when increasing the ratio between areas ($\chi_{1}^{2}$ = 18.976, *p* < 0.001). Males had significantly higher performances compared to females ($\chi_{1}^{2}$ = 6.024, *p* = 0.014) and there was no difference between zebrafish trained to select the larger or the smaller area ($\chi_{1}^{2}$ = 2.749, *p* = 0.097). No interaction was statistically significant (all *p* values > 0.160).

The comparison of the log-likelihoods of models with and without the random effect revealed no inter-individual difference in numerical discrimination (*p* = 0.998). The same result was obtained analysing each ratio separately (all *p *values > 0.205).

### Comparison between numerical and continuous quantity discriminations

The overall analysis revealed a significant improvement in subjects’ accuracy over training session (GLMM: $\chi_{1}^{2}$ = 26.071, *p* < 0.001), and a significant decrease in subjects’ accuracy when increasing the ratio between quantities ($\chi_{1}^{2}$ = 103.209, *p* < 0.001). Zebrafish had significantly higher performances when trained to discriminate between areas than between numerosities ($\chi_{1}^{2}$ = 7.270, *p* = 0.007). No interaction was statistically significant (all *p* values > 0.322).

### Absolute versus relational discrimination

Zebrafish trained on numbers significantly chose the previously reinforced quantity in probe trials presenting two areas either considering the first choice (65.63 ± 12.50%; one sample *t* test, *t*_15_ = 21.002, *p* < 0.001) or the time spent near the reinforced stimulus (62.39 ± 8.07%; *t*_15_ = 30.926, *p* < 0.001). Performance in probe trials did not differ between the three levels of control for cumulative surface area ($\chi_{2}^{2}$ = 2.001, *p* = 0.368). Zebrafish trained on areas significantly chose the previously reinforced quantity in probe trials presenting two numerosities either considering the first choice (63.28 ± 14.77%; one sample *t* test, *t*_15_ = 17.140, *p* < 0.001) or the time spent near the reinforced stimulus (64.27 ± 9.79%; *t*_15_ = 26.252, *p* < 0.001).

Fish trained on numbers and controls trained on areas did not differ in accuracy of probe trials (GLMM: $\chi_{1}^{2}$ = 0.308, *p* = 0.579).

## Discussion

### Numerical discrimination

The capacity to discriminate numerosities has been investigated in many fish species, but only a few studies have carefully controlled stimuli for the continuous perceptive variables that covary with number, and only one teleost species, the guppy, was studied with task difficulties that allow a direct comparison with mammals and birds. In the present study, we investigated this issue in another teleost, the zebrafish. To determine numerical acuity, male and female zebrafish underwent a series of numerical discrimination tasks of increasing difficulty. Contrary to previous evidence (Agrillo et al. 2012; Seguin and Gerlai 2017), zebrafish demonstrated excellent numerical abilities. All subjects rapidly learned numerical discriminations up to 3 versus 4 items. Most zebrafish, 10 out of 16, also learned the 4 versus 5 discrimination but none of them reached learning criterion in the 5 versus 6 task. Interestingly, when we analysed this task at the group level, we found performance significantly above chance, suggesting that 5 versus 6 might represent the threshold of numerosity discrimination of this species.

We found no difference in performance between trials in which stimuli were totally or partially controlled for the cumulative surface area or between trials controlled for density and those controlled for convex hull (i.e. the smallest polygon enclosing all items). This indicates that zebrafish did not use these three non-numerical cues to solve the task. Although it has never been demonstrated in any species and evidence is conflicting for infants (Starr and Brannon 2015), in principle, it is possible that a species uses the sum of contours of the items as a proxy for number. Cumulative surface area and cumulative contour length cannot be simultaneously controlled for unless different shapes are used for the larger and smaller number. However, the control for area in this study also provided a partial control for perimeter, and analysis conducted only on trials in which the total perimeter was controlled showed even this cue was likely not used by zebrafish to solve the task. We also found no difference in performance between subjects trained on the smaller or the larger numerosity or between males and females.

Zebrafish have shown high acuity in discrimination of areas (Santacà et al. 2020a, b; control experiment of this study). In theory, they could have solved numerical tasks without using numerical information, if they had responded correctly to two-thirds of the stimuli that were only partially corrected for the area. Conversely, they were found to select the correct stimulus significantly often, even in the subset of stimuli in which the larger numerosity had the smaller cumulative area. This confirmed that zebrafish, like some higher vertebrates (Cantlon and Brannon 2007; Wagener et al. 2018), spontaneously attended to the numerical attributes of stimuli even when non-numerical cues were available as an alternative to solve the task.

It was suggested that vertebrates possess two distinct numerical systems operating over different portion of the numerical range (Feigenson et al. 2004). One, the small number system, is precise but subject to a set size limit of 4 items; the other, the approximate number system, has no upper limit but is ratio dependent. The present study seems to support this hypothesis because the performance is similar for the first three ratios tested (up to 3 versus 4) and progressively decreases from 3 versus 4 to 5 versus 6. However, it is difficult to draw firm conclusions on this issue because numerical tasks were presented to all subjects with the same order, from the easiest to the most difficult and there might have been carry-over effects from prior tests on subsequent.

When comparing the results of this experiment with an identical experiment conducted with guppies (Gatto et al. 2021), no difference appeared in the learning rate between the two species. However, in zebrafish but not in guppies, the performance in the 5 versus 6 discrimination task was above chance at the group level, an indication that numerical acuity might be slightly better in the former species.

Despite the high numerical acuity shown by zebrafish and guppies (Gatto et al. 2021), which places them close to the most encephalized vertebrates, a difference remains with the results of the studies conducted on the latter species. In both guppies and zebrafish, accuracy did not exceed 75% correct even in the easier numerical tasks, which is considerably less than the 90–95% accuracy commonly observed in studies on mammals and birds (Cantlon and Brannon 2007; Roberts and Mitchell 1994). One possibility is that large brained animals can reach a greater accuracy in discrimination learning tasks. On the other hand, the disparity may also be due to differences in method. Studies on birds and mammals usually involve thousands of reinforced trials (Cantlon and Brannon 2007; Jaakkola et al. 2005; Roberts and Mitchell 1994), whereas in each numerical task, zebrafish and guppies were allowed a maximum 120 trials and the task ended when they reached the 75% learning criterion. In favour of the latter hypothesis, DeLong et al. (2017), after training goldfish to a 0.66 discrimination (2 verses 3 and 10 versus 15) for 1200 trials, found that their performance exceeded 90% accuracy.

The results of this study may be of some relevance for translational research. Zebrafish have recently become a popular model organism in numerous areas of neurobiological research, including the investigation of human brain diseases (Leung et al. 2013; Norton 2013; Paquet et al. 2009). The discovery that zebrafish have numerical capabilities comparable to other teleosts and similar to those observed in higher vertebrates opens the way to the possibility of using this model species to study the neural basis of numerical cognition of vertebrates and to identify the genetic underpinnings of human developmental disorders such as dyscalculia.

### Discrete versus continuous quantity discrimination

The results of the control experiment on discrimination of areas confirmed the excellent quantificational abilities of zebrafish. The overall trend is very similar to that observed in the numerical experiment with the exception that here four males and one female reached the learning criterion in the 0.83 ratio (corresponding to 5 versus 6 in the numerical discrimination task). None of these five subjects reached the learning criterion in the 0.86 ratio task, but an overall analysis indicated that their performance was above chance level. Indeed, a direct comparison of the learning performance of the two groups showed zebrafish were significantly more accurate in discriminating areas than they were in discriminating discrete quantities. The performance shown by zebrafish in this study with an appetitive conditioning paradigm confirms the results shown by this species using a more natural setting and a spontaneous preference paradigm (Santacà et al. 2020a, b). The study exploited the natural tendency of fish to pass through the largest hole to measure the capacity of zebrafish to discriminate between two holes of different size. Zebrafish significantly discriminated all area ratios from 0.60 to 0.91, although in the most difficult ratios (0.86 and 0.91), the rate of correct choices was just over 50% as observed in the present study.

It is interesting to note that in this control group, we found a difference between the sexes with a slightly but significantly higher performance in males. In teleosts, sexual differences have so far been found in various cognitive functions, sometimes in favour of males, others in favour of females; although some hypotheses have been put forward, the reason these differences exist is presently unclear (Lucon-Xiccato and Bisazza 2017; Triki and Bshary 2021; Wallace and Hofmann 2021). The sexual difference observed here might be related to sexual selection mechanisms. In teleosts, female fecundity increases with body size, and due to allometry, a small variation in body length determines a large difference in the number of offspring produced. As with many other teleosts (Hoysak and Godin 2007; Sargent et al. 1986), male zebrafish may have evolved mate choice mechanisms associated with the capacity to perceive subtle body size differences among prospective mates, a mate preference that instead does not seems to occur in female zebrafish (Spence and Smith 2006).

In contrast with studies conducted on other teleosts (Lucon-Xiccato and Bisazza 2017; Mair et al. 2021; Miletto Petrazzini and Agrillo 2016), our experiments provided no evidence of inter-individual differences in performance on cognitive tasks. It was suggested that in animals a large part of individual differences in cognitive performance might be attributable to non-cognitive factors such as personality or motivation (Carere and Locurto 2011; Sih and Del Giudice 2012). In some fish, the degree of adaptation to the experimental procedure has a dramatic effect on cognitive performance, and individual differences in the capacity to acclimate are more evident for some procedures than they are for others (Gatto et al. 2021). The procedure adopted in this study, which involved testing the subjects in their own tank and progressively adapting them to the task, may have minimized the role of non-cognitive factors on performance. This hypothesis is indirectly supported by the fact that unlike many previous studies (Bisazza et al. 2014; Gatto et al. 2021; DeLong et al. 2017; Lucon-Xiccato et al. 2015), none of the fish selected for this study needed to be replaced due to poor adaptation to experimental conditions.

Why are zebrafish so efficient in both tasks? On the one hand, it is possible that both capabilities are highly adaptive in nature. The ability to estimate the size of a hole is very important and one fish that makes an inaccurate estimate could be hurt or become stuck and consequently captured by a predator. The ability to gauge dimensions could also be very important for accurately estimating the size of a rival, a potential sexual partner, or prey (Earley et al. 2003; Quinney and Ankney 1985; Rosenthal and Evans 1998). Many advantages can also be hypothesized for the discrimination of numbers. For instance, fish that choose a school of five instead of four fish, due to a dilution effect and increased vigilance, are much less likely to be caught when a predator arrives (Krause and Ruxton 2002). Other benefits can be the possibility of choosing the largest number of individuals of the opposite sex or the largest group of prey (Agrillo et al. 2008a; Wellenreuther and Connell 2002).

Alternatively, some authors have suggested vertebrates may have a single quantificational system that presides over various types of estimation of continuous and discrete quantities (i.e., numerosity), irrespective of dimensions (e.g., areas, distances, duration of events), the so-called ATOM theory (Agrillo and Miletto Petrazzini 2013; Walsh 2003). The theory currently does not have strong support in humans or higher vertebrates and has received very little attention in fish (Agrillo and Miletto Petrazzini 2013; Miletto Petrazzini and Brennan 2020).

### Absolute versus relational discrimination strategy

There are two strategies to learn a numerosity discrimination task. The first consists of learning that one numerosity is correct and the other incorrect (absolute value strategy). The second consists of learning to respond to the larger (or the smaller) of the two quantities irrespective of their absolute value (relational strategy). The former strategy requires that the subject discriminate a specific numerosity from all the others, the latter that the subject develops the relational concept of larger and smaller. For instance, honeybees (*Apis mellifera*) were observed to solve a numerical task using an absolute strategy (Bortot et al. 2019); on the other hand, pigeons (*Columbia livia*) trained to discriminate between two numbers readily transferred the learned rule to different numerosities (Honig and Stewart 1989).

To gain insight into the strategy used by zebrafish, at the end of the experiment, fish trained on numbers were tested in unrewarded trials on areas and those trained to discriminate areas were given the choice of two numerosities. Zebrafish trained on numbers spontaneously generalize to areas, indicating this species learned numerical discriminations using a relational strategy. The spontaneous use of a relational strategy was observed in other fish species. Miletto Petrazzini and collaborators (2016) trained angelfish to select a stimulus containing 10 dots (in either 5 versus 10 or 10 versus 20 comparisons). When tested in probe trials with the previously trained numerosity and a novel one (respectively, 20 or 5), subjects selected the novel numerosity showing that they had learned the task using a relative rule. Remarkably, human adults trained as the angelfish (i.e., in absence of explicit verbal instructions) also proved to spontaneously use a relational strategy to solve numerical discrimination tasks. The use of a relational strategy was demonstrated in another teleost, the guppy (Miletto Petrazzini et al. 2015a, b). Another experiment of the same study investigated whether guppies could also learn an absolute strategy, if required by the task. These subjects were trained to select one specific numerosity, 4, against several alternatives (i.e., 4 versus 1, 4 versus 2, 4 versus 8, and 4 versus 10). Guppies not only proved able to learn to recognize the number 4 against all alternatives but also generalized the acquired discrimination to novel, more challenging numerical contrasts (4 versus 3 and 4 versus 6).

Interestingly, in our study, controls trained to select the larger (or smaller) figure spontaneously selected the larger (or smaller) numerosity even though, due to control for cumulative surface area, the larger numerosity contained on average smaller dots (and vice versa). Thus, zebrafish that learned the relational concept of larger and smaller in a non-numerical context seem to transfer the learned rule spontaneously to the number of items rather than to the size of the individual items.

The capacity to learn relational concepts and apply them to a different context has recently been demonstrated in angelfish (Miletto Petrazzini and Brennan 2020). The experiment was similar to that of the present study. Some angelfish, initially trained to select the smaller numerical quantity, spontaneously transferred the learnt rule to continuous quantities, selecting the shorter of two lines. Conversely, fish trained on a line-length discrimination task spontaneously generalized the learnt rule to numbers. The transfer of the rules learned in a numerical context to continuous quantities and vice versa might be facilitated in vertebrates by the existence of the supposed common brain network for the representation of time, space, number and other magnitudes that we have mentioned above (Agrillo and Miletto Petrazzini 2013; Walsh 2003).

### Numerical abilities of teleosts

In vertebrates, numerical acuity appears to correlate with the size and degree of complexity of nervous system. Cartilaginous fish are very poor at discriminating numerical quantities, amphibians and reptiles show rudimentary numerical discrimination; numerical abilities become increasingly more sophisticated in mammals and birds, approaching those typical of humans in large-brained species such as primates and corvids (Agrillo 2015; Agrillo and Bisazza 2018; Khatiwada and Burmeister 2021; Kreuter et al. 2021; Szabo et al. 2021). The idea of a relationship between brain complexity and accuracy in numerical discrimination is further reinforced by the observation that during human ontogeny numerical acuity gradually increase from birth to early adulthood (Coubart et al. 2014; Halberda and Feigenson 2008).

In teleosts, numerical abilities have been studied in great detail in the guppy (Bisazza et al. 2014; Lucon-Xiccato et al. 2017) and in a closely related species, the mosquitofish (Agrillo et al. 2010, 2012). Like some mammals and birds, these species have been shown to discriminate large numerosities with a numerical ratio effect but apparently without an upper limit (e.g., 100 from 200 items) and accurately discriminating small numerosities for example 5 from 6 conspecifics (Agrillo et al. 2010, 2012; Lucon-Xiccato et al. 2017). Guppies were also shown to use ordinal information (e.g., learn to recognize the third feeder in a row of eight identical ones) and mosquitofish to exhibit some proto-arithmetical ability, being able to evaluate the numerosity of an array even when they could see only one item at a time (Dadda et al. 2009; Miletto Petrazzini et al. 2015a, b).

Here we have shown that numerosity discrimination capacities of zebrafish appear equal, if not slightly superior, to those found in guppies in an identical experiment. The two species are representative of the two large clades (Acanthopterygians and Ostariophysians) that comprise the majority of teleosts and that diverged more than 200 million years ago (Steinke et al. 2006). This study thus reveals that, contrary to previous suggestions, guppies and their relatives are not an exception and that sophisticated numerical skills may be a common feature of teleosts. It remains to be understood why teleosts show numerical capacities more advanced than those of chondrichthyans, amphibians, reptiles and some birds and mammals are, and not too different from those species, corvids, parrots and non-human primates that have evolved the highest cognitive capacities.

One possibility is that possessing numerical abilities is more important for fitness in teleosts than it is in other taxa. For example, the majority of teleosts spend a large portion of their lives in groups and, as shown by many studies, the size of the group strongly affects various fitness-related functions such as foraging, anti-predator defence, mating and reproduction (Mariette et al. 2010; Milinski 1979; Taborsky et al. 2005; Pitcher 1986). With rare exceptions this does not happen in cartilaginous fishes, amphibians and reptiles; many mammals are solitary or live in family groups and most birds are gregarious only at certain times of the year, for instance, in winter flocking or in breeding colonies where counting the conspecifics does not appear to be essential (Krause and Ruxton 2002; Wilson 2000).

On the other hand, some higher order cognitive functions once believed to be unique to some mammalian and avian species have now been found in teleost fish in spite of their relatively small brain size. For instance, some fish species use tools, learn new habits from experienced conspecifics, show innovative problem solving, learn complex spatial mazes, and display episodic-like memory (Brown 2012; Brown and Laland 2003; Brown et al. 2008; Hamilton et al. 2016; Mair et al. 2021). An explanation for this intriguing finding is that after the divergence from lobe-finned fishes (the lineage that gave rise to tetrapods), teleosts underwent a whole-genome duplication, an event which is thought to have played a major role in promoting diversification and evolutionary innovation in this group of vertebrates (Glasauer and Neuhauss 2014; Ravi and Venkatesh 2018). It is possible that this also favoured the evolution of novel and complex cognitive functions (Roux et al. 2017; Yamamoto and Bloch 2017). In support of this claim, Schartl et al. (2013) found that in teleosts, duplicates of genes that code for brain functions have been retained more often than protein-coding genes involved in well conserved functions have (e.g., liver genes). Data from a larger number of fish species are clearly needed to test these hypotheses and to reconstruct the evolutionary origin of sophisticated numerical abilities of this lineage. The pool should comprise fish with different ecologies and life history strategies as well as species from different taxonomic groups, including basal ray-finned fishes (e.g., eels and sturgeons), lobe-finned fishes (e.g., lungfish), and cartilaginous fishes (e.g., shark and rays) (Agrillo and Bisazza 2018; McCluskey and Braasch 2020; Yamamoto and Bloch 2017).

It must also be said that discriminating the larger and the smaller of two quantities of objects is only one of the many numerical functions, not necessarily among the most sophisticated. For example, some mammals and birds can represent numbers abstractly and match numbers of events across sensory modalities as well as learn to use Arabic numerals to represent a precise quantity or add and subtract quantities (Biro and Matsuzawa 2001; Jordan et al. 2008; Rugani et al. 2019). Though there are indications that fish might possess rudimentary forms of these functions (Dadda et al. 2009; Miletto Petrazzini et al. 2015a, b), a challenge for future research is to determine the extent to which the numerical capabilities of fish compare with those of warm-blooded vertebrates.

## Supplementary Information

Below is the link to the electronic supplementary material.

Supplementary file1 (DOCX 401 KB)

## Declarations

### Availability of data and material

The datasets generated during the current study are available from the corresponding author on reasonable request.
