# Supplementary material for: Zebrafish excel in number discrimination under an operant conditioning paradigm
Source: Anim Cogn. 2022 Feb 18;25(4):917–33. doi: 10.1007/s10071-022-01602-y (PMC9334370; doi:10.1007/s10071-022-01602-y)
Supplement: Supplementary file 1 — Supplementary file1 (DOCX 401 KB) [file 10071_2022_1602_MOESM1_ESM.docx]

**Numerical discrimination in zebrafish**

Angelo Bisazza and Maria Santacà

**Additional methods**

All experiments were performed in accordance with the European Legislation for the Protection of Animals used for Scientific Purposes (Directive 2010/63/EU) and the Italian animal-protection standards (D.lgs. 26/2014).

1. Subjects and animal housing

Overall, 32 adult zebrafish were used in this study: 16 (8 males and 8 females) in the numerical discrimination experiment and 16 (8 males and 8 females) were used in the continuous quantity discrimination. No subject was replaced during the experiment.

1. Apparatus

Subjects were individually maintained for the duration of the experiment in hourglass-shaped experimental apparatuses (Figure S1). Stimuli were inserted in the short wall of the tank after the subject spontaneously moved in the opposite side of the tank to avoid any stress that could affect subject’s performance. Externally the two long sides of each tank were covered with opaque green plastic sheets (thickness: 3.5 mm; Ecoplak). To increase the visibility of the stimuli, the inside of both short walls of each apparatus was covered with white waterproof polyester sheets (thickness: 260 µm; Picofilm, Sihl). Each tank was provided with natural polychrome gravel (2-5 mm), plants (*Hygrophila corymbosa*) and two mirrors were placed in two lateral compartments.


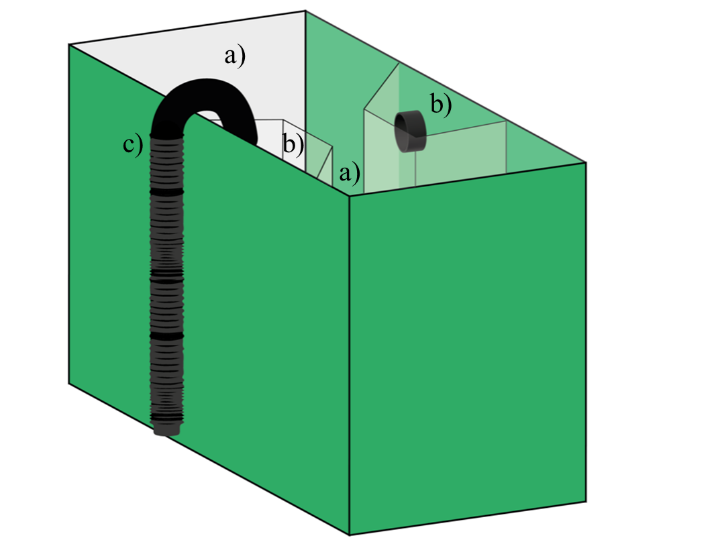


Figure S1 Individual training apparatus. A) Hourglass-shaped testing compartment B) Lateral compartments housing plants and mirrors C) Water from aquarium housing conspecifics.


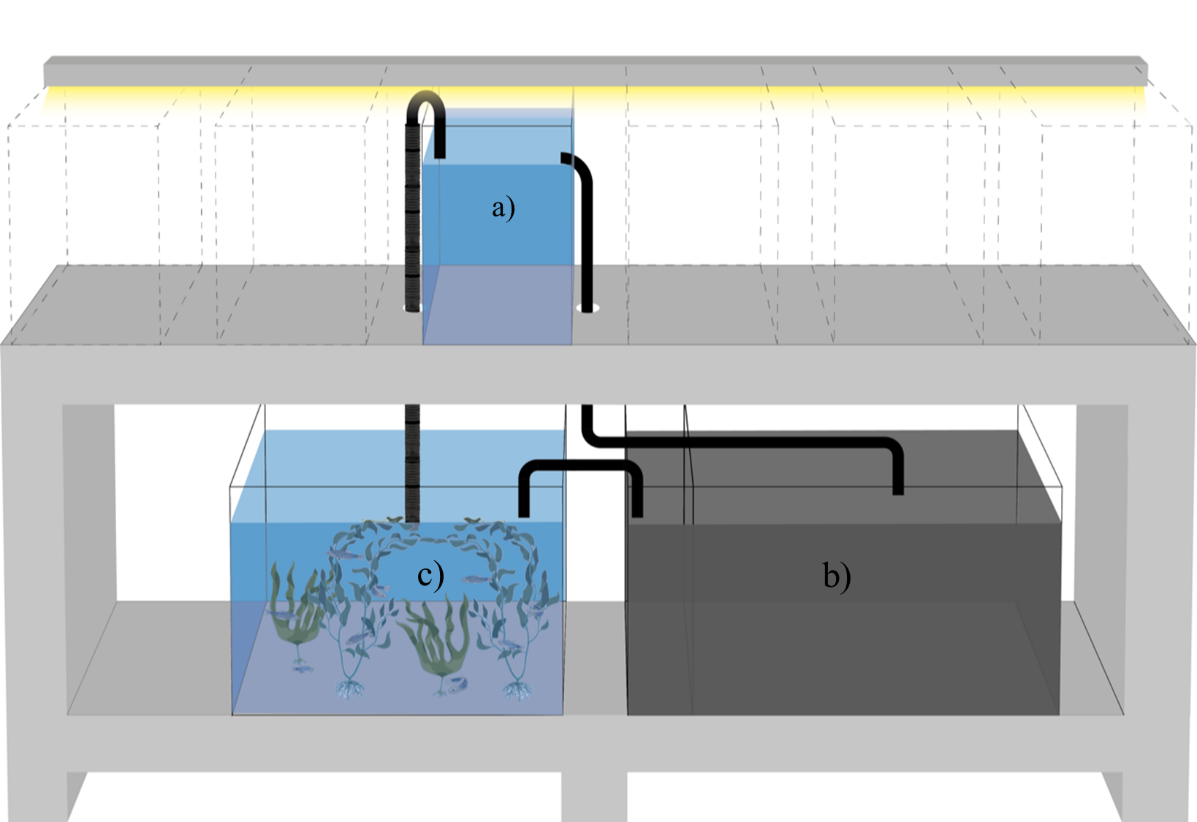


Figure S2. Recirculating water system for filtering and providing olfactory social cues to the subjects. A) individual tank; B) Biomechanical filter; C) Aquarium housing conspecifics.

A recirculating water system (Figure S2) connected each individual tank to a biomechanical filtration system (one filtering system every six tanks). Filtered water was brought into a large aquarium with gravel bottom and abundant vegetation, which housed a social group (approx. 20 adult fish). Water was then pumped from the aquarium back to the individual tanks (flow rate approx. 30 l/h). Biomechanical filters were controlled periodically. Water quality checks were performed weekly. We checked for these parameters: temperature, water acidity (pH test), carbonate hardness (KH test), total hardness (GH test), toxic nitrogen compounds (nitrites and nitrates). Additionally, we refilled with deionized water twice a week and a veterinarian checked fish health once a week. Each group of six tanks was lit by a 36W fluorescent lamp leaning over them.

(c) Stimuli

In the numerical discrimination, stimuli consisted of sets of black dots (diameter range 0.75–0.95 cm) on a white background. We used 24 different pairs of stimuli for each ratio that were controlled for the cumulative surface area (76 to 85% in one-third, 86 to 95% in one-third, and 96 to 105% in the final one-third), the convex hull and the density of the dots (Figure S1). Additionally, the control for cumulative surface area also produced a partial control for cumulative contour length (79 to 85%, 86 to 95% and 96 to 105%).


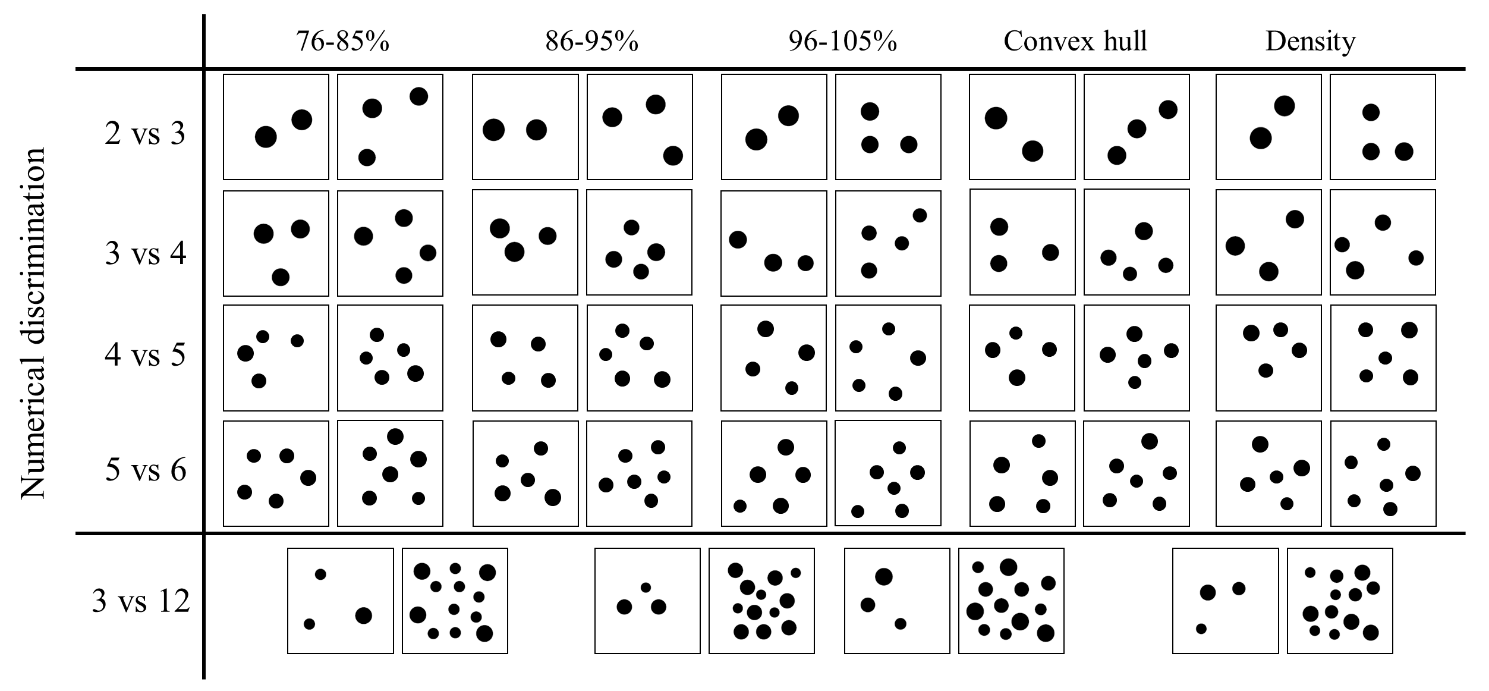


**Figure S3. Experimental stimuli.** Examples of stimuli for each ratio used in the numerical discrimination that were controlled for the three levels of cumulative surface area (76-85%, 86-95% and 96-105%), the convex hull and the density of the dots. The 3 versus 12 stimuli were not controlled for non-numerical variables.

(d) Analisys of videorecordings

*Interrater reliability.* All trials were recorded with video cameras (Canon LEGRIA HFR38) placed above each tank. To score subjects’ accuracy, we played back one-third of the recordings of each ratio for each subject on a computer using a VLC media player (Videolan, [**https://www.videolan.org/vlc/index.html**](https://www.videolan.org/vlc/index.html)) noting the chosen stimulus. We considered a choice when the subject approached (swam at less than 1 body length) one stimulus.

*Scoring of absolute versus relational discrimination test.* To score subjects’ time spent near each stimulus (within one body length) in the unreinforced trials, we used a computer software originally developed in our laboratory (‘Ciclic Timer’, written in Delphi 5 Borland).

(e) Additional statistical analysis on density and convex hull

To further investigate the influence of density or convex hull on the subjects’ performances, we split our dataset into the trials that controlled for density and those that controlled for convex hull and for each set we performed a generalized mixed-effects model for binomial distributions (GLMM) with the reinforced stimulus, the training session, the sex and the ratio as fixed effects, and the individual ID as a random effect. Analyses were performed in R version 4.0.5 (The R Foundation for Statistical Computing).

**Additional results**

*Interrater reliability.* Interrater reliability was calculated with Cohen’s kappa coefficient and was found to be very high for numerical (κ = 1, *p* < 0.001), size (κ = 1, *p* < 0.001) and absolute versus relational discrimination (κ = 0.998, *p* < 0.001).

*Density and convex hull analyses.* Both GLMMs confirmed the results of the main model conducted on the whole set of trials. The GLMM considering only the trials that controlled for density revealed a significant improvement in subjects’ accuracy over training session (GLMM: χ^2^_1_ = 19.274, *p* < 0.001), and a significant decrease in their accuracy when increasing the ratio between numerosities (χ^2^_1_ = 37.581, *p* < 0.001). We found no difference between males and females (χ^2^_1_ = 0.714, *p* = 0.398) or between zebrafish trained to select the larger numerosity or the smaller numerosity (χ^2^_1_ = 0.987, *p* = 0.320). No interaction was statistically significant (all *p*-values > 0.158). The GLMM considering only the trials that controlled for convex hull revealed a significant improvement in subjects’ accuracy over training session (GLMM: χ^2^_1_ = 24.572, *p* < 0.001), and a significant decrease in their accuracy when increasing the ratio between numerosities (χ^2^_1_ = 21.857, *p* < 0.001). We found no difference between males and females (χ^2^_1_ = 1.347, *p* = 0.245) or between zebrafish trained to select the larger numerosity or the smaller numerosity (χ^2^_1_ = 2.572, *p* = 0.109). No interaction was statistically significant (all *p*-values > 0.395).
